# Collembola laterally move biochar particles

**DOI:** 10.1371/journal.pone.0224179

**Published:** 2019-11-01

**Authors:** Stefanie Maaß, Ronja Hückelheim, Matthias C. Rillig

**Affiliations:** 1 University of Potsdam, Institut für Biochemie und Biologie, Plant Ecology and Nature Conservation, Potsdam, Germany; 2 Berlin-Brandenburg Institute of Advanced Biodiversity Research (BBIB), Berlin, Germany; 3 Freie Universität Berlin, Institut für Biologie, Plant Ecology, Berlin, Germany; RMIT University, AUSTRALIA

## Abstract

Biochar is being discussed as a soil amendment to improve soil fertility and mitigate climate change. While biochar interactions with soil microbial biota have been frequently studied, interactions with soil mesofauna are understudied. We here present an experiment in which we tested if the collembolan *Folsomia candida* I) can transport biochar particles, II) if yes, how far the particles are distributed within 10 days, and III) if it shows a preference among biochars made from different feedstocks, i.e. pine wood, pine bark and spelt husks. In general, biochar particles based on pine bark and pine wood were consistently distributed significantly more than those made of spelt husks, but all types were transported more than 4cm within 10 days. Additionally, we provide evidence that biochar particles can become readily attached to the cuticle of collembolans and hence be transported, potentially even over large distances. Our study shows that the soil mesofauna can indeed act as a vector for the transport of biochar particles and show clear preferences depending on the respective feedstock, which would need to be studied in more detail in the future.

## Introduction

Biochar has received much attention as a potential means to mitigate climate change via the sequestration of carbon, but it may also be useful for improve soil fertility (e.g. [1]; [2]; [3]; [4]; [5] for review). The latter effect has been shown to be related to an increase in soil pH ([6]), and may also improve nutrient retention ([7]). There have been several studies showing that biochar has the potential to change soil biological communities in regard to their composition and abundance ([8]; [9]; [10]; [11]; [12]; [13]; [14]; [15]). Also, there is evidence that biochar can reduce the infection rate of nematodes causing root-lesions in carrots ([16]), increases soil microbial biomass ([11]; [14]Ji; [12]) and reduce or not affect colonization of roots by arbuscular mycorrhizal fungi ([17]; see [18] for extensive review). Although there has been intense research in terms of the effect of biochar amendments on soil microorganisms and earthworms and their respective interactions ([18]), the two most abundant groups of mesofauna, i.e. Collembola (springtails) and Acari (mites), have received less attention ([19]). As these groups are partly part of the fungal energy channel in the soil food web ([20]), one should expect a close interaction with microbial populations ([18]). In addition, there is little evidence of how the mesofauna contributes to the distribution of biochar in the soil, which might be especially important in terms of the long-term persistence of biochar amendments in the soil. Presumably, the distribution happens via i) attachment of particles on setae or cuticle; ii) feeding and defecation elsewhere; iii) animal movement over particles and hence pushing ([21]). However, Gormsen et al. ([22]) showed that Collembola are indeed able to act as vectors for fungal spores, and other studies support this activity for charcoal ([23]; [24]), hydrochar ([19]; [25]; [26]) and microplastics ([21]). As biochars of different feedstocks show different characteristics, we wanted to determine if the collembolan *Folsomia candida* I) can transport biochar particles, II) if yes, how far the particles are distributed within 10 days, and III) if it shows a preference among biochars made from different feedstocks. To test these questions, we conducted an arena experiment with three biochars originating from different feedstocks: pine wood, pine bark and spelt husks.

## Material & methods

We used three biochars originating from different feedstocks: pine bark and pine wood of *Pinus sylvestris* (PB and PW, respectively) and spelt husks (SH). These biochars were produced for the experiment of George et al. ([16]) and stored in glass bottles until we used it for our experiment. All feedstocks were air-dried at room temperature prior to carbonization. The initial feedstocks were covered with sand and then wrapped in aluminum foil to create an atmosphere reduced in oxygen necessary for carbonization, which lasted for five hours at 500°C (highest treatment temperature) in a muffle oven. The carbonized material was then sieved to separate it from the sand. Each biochar was crushed with a hammer and then sieved to a particle fraction of 100–200 μm, which we used for the experiment. We decided to use this particle fraction because it has proven to be the optimal size for *F*. *candida*-*‒*mediated potentially transport in previous experiments ([21]) and is still countable on photos for later quantitative analysis. Details about each biochar’s properties such as water-holding capacity and nutrient concentrations have been reported elsewhere ([16]). Experimental units were 10-cm-diameter specimen cups filled with a 1-cm layer of plaster of Paris which was wetted to saturation. Treatments consisted of 2 mg of the respective biochar type distributed in a 0.5-cm-diameter circle (‘feeding station’) in the middle of the cups. We did not offer any additional food source. To avoid airflow that could potentially distribute the biochar particles, we carefully placed lids on the specimen cups. They were stored at room temperature (20°C ± 2°C) during the experiment.

The target organism was *Folsomia candida* (Collembola) with a body size of up to 3 mm ([27]) from our laboratory cultures, originating from Northern Germany. The individuals were kept on a Plaster of Paris—activated charcoal mix and fed with Baker’s yeast before starvation which started 14 days prior to the experiment. We set up 8 replicates of each treatment with 15 *F*. *candida* per cup. Controls duplicated the *F*. *candida* treatments but did not contain any Collembola, resulting in a total of 48 samples.

**Fig 1:**
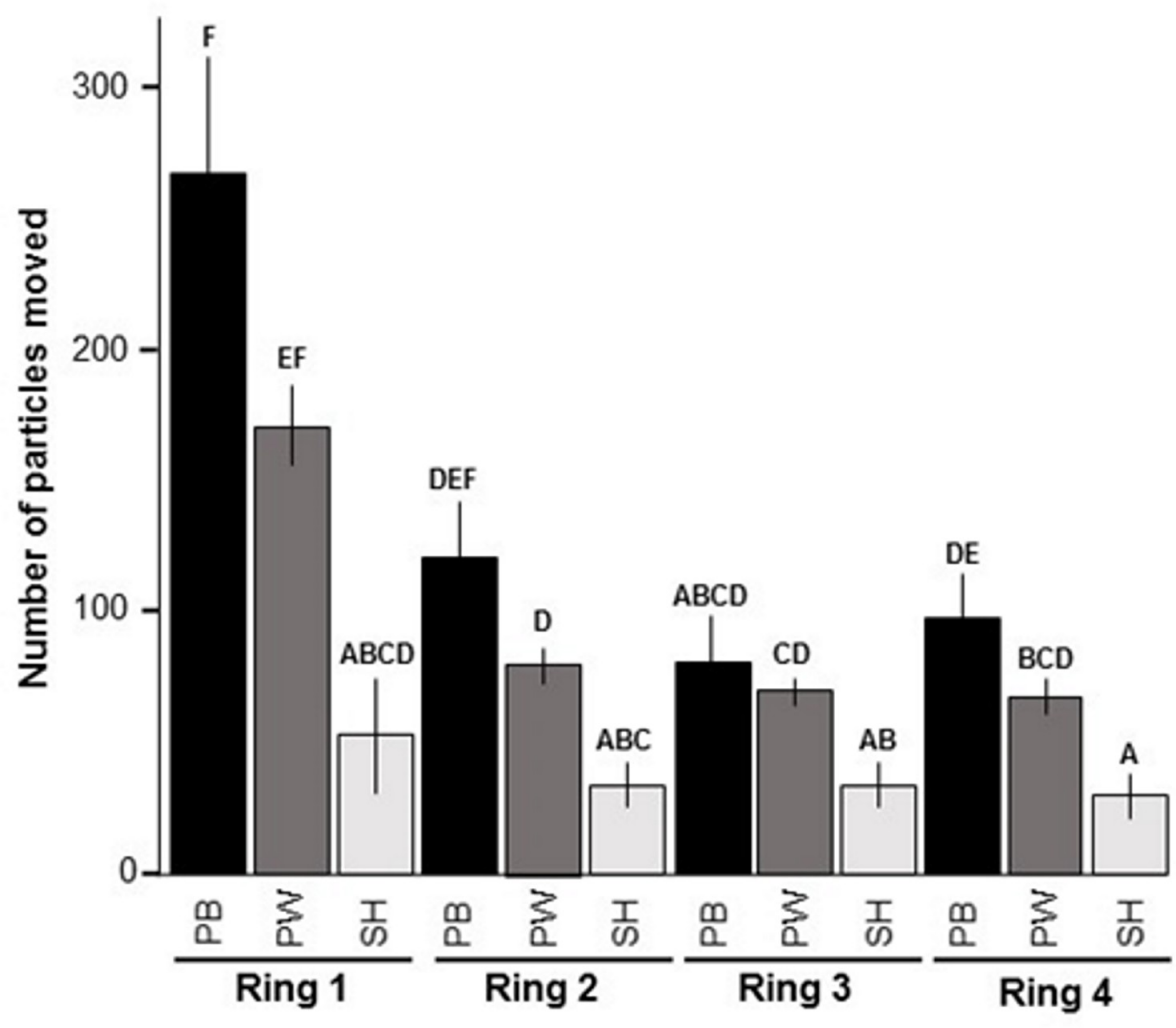
Number of particles moved horizontally over the four defined rings (with ring 1 = 1 cm, ring 2 = 2 cm, ring 3 = 3 cm and ring 4 = 4 cm diameter) around the feeding station by *Folsomia candida* at the end of the experiment (after ten days). The shading of bars represents the different biochar types used (black = PB, pine bark; dark grey = PW, pine wood; light grey = SH, spelt husks). Mean ± SE, n = 8. Bars with same letters are not significantly different according to pairwise comparisons of least square means at alpha level 0.05. Controls were 0, hence not shown.

For ten days, each sample was photographed once a day from a distance of 30 cm. For the analysis of the images, four concentric circles of 1, 2, 3 and 4 cm diameter (corresponding to ring 1, ring 2, ring 3 and ring 4, see Fig 1) were digitally placed around the feeding station and the particles in each ring were counted ([21]).

### Data analysis

For the analysis of the data we used R, version 3.3.1 ([28]). We used generalized least square models of the ‘nlme’ package ([29]) and used the function ‘varIdent’ to account for heterogeneity in our data ([30]). We checked the model residuals for normal distribution and homogeneity of variances. Pairwise comparisons of least square means of factors were performed by the package ‘lsmeans’ ([31]). For generating the figures, we used ‘ggplot2’ ([32]).

## Results

We found highly significant differences between the rings, i.e. distance of particle transport (F_1_ = 14.29, p <0.001), particles, i.e. biochar types (F_1_ = 36.73, p < 0.001) and a significant interaction term for ring and particle (F_1_ = 2.51, p = 0.03) (see Table 1).

**Table 1:** Results of two-factors ANOVA (ring / horizontal distance and particle / biochar type). Significant p-values <0.05 shown in bold.

	df	F	p
(Intercept)	1	561.5481	**<0.001**
ring	3	14.2803	**<0.001**
particle	2	36.7340	**<0.001**
ring : particle	6	2.5131	**0.0276**

In general, biochar particles from the feedstocks pine bark and pine wood (PB and PW, respectively), were consistently distributed significantly more than those made of spelt husks (SH, Fig 1).

**Fig 2:**
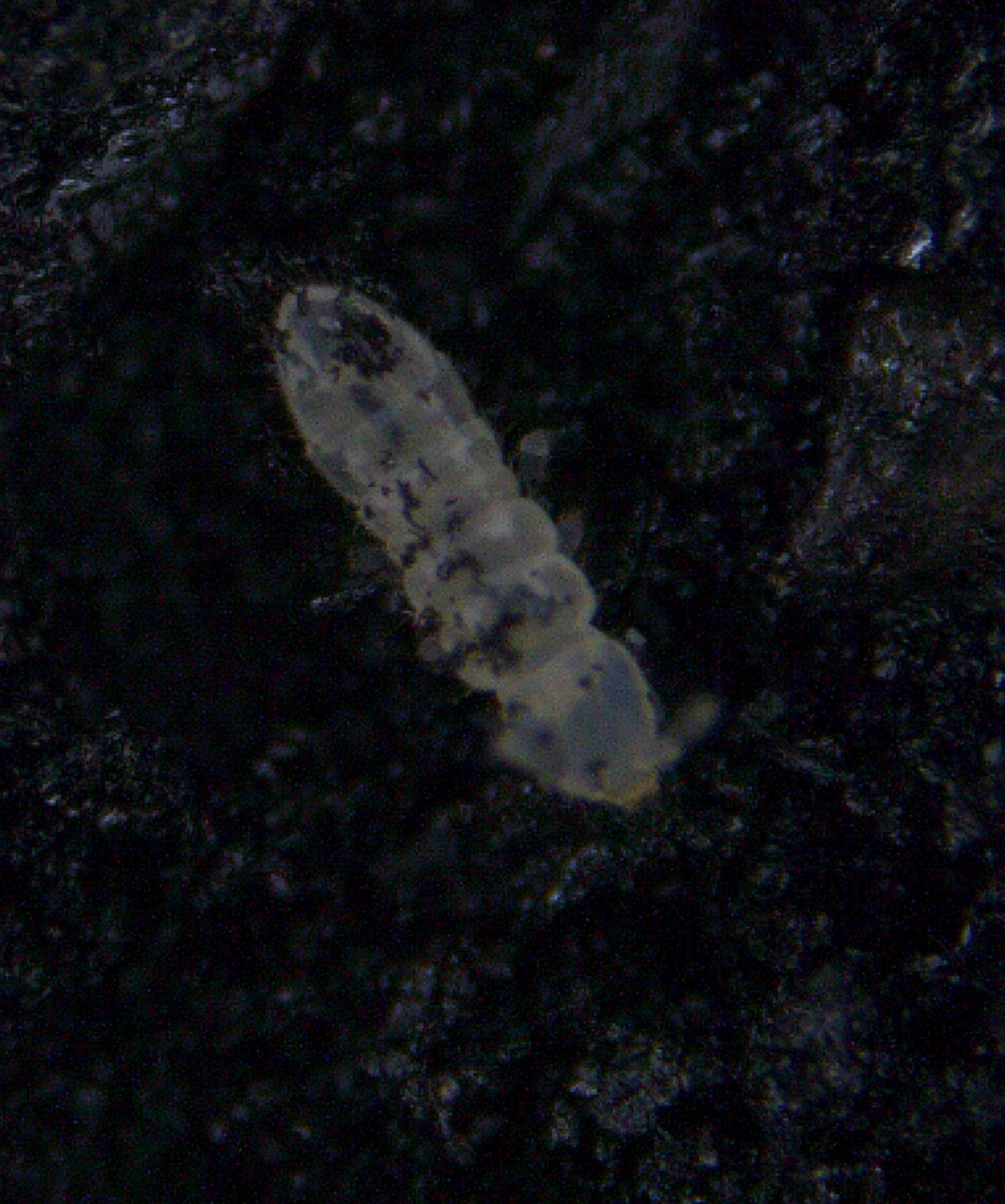
*Folsomia candida* individual covered with pine bark biochar particles and biochar particles in the gut.

Additionally, we found *Folsomia candida* individuals which, after jumping into the pine bark particles, were covered with a large number of particles that remained on them even when the animals left the pile of biochar (Fig 2). In addition, we observed in nearly every collembolan black dots in the gut, indicating that they ingested microparticles by grazing the surface of larger biochar particles and hence could defecate them somewhere else ([23]; [24]; [19]; [15]).

## Discussion

The transport of objects has been much-studied in soil macrofauna such as earthworms. However, there is evidence that the highly abundant soil mesofauna is involved in the transport of small particles such as fungal spores, microplastics and hydrochar (e.g. [22]; [21]; [19]; respectively). We tested the ability of a springtail species to distribute biochar particles of various feedstocks and whether transport occurred differentially among the three biochars. Indeed, all three biochar types were transported more than 4 cm within the experimental time span; however, there were clear differences in how frequently and far the biochar types were transported: biochar based on pine bark (PB) was transported most, followed by pine wood and spelt husk biochar.

Biochar particles can be attached to the setae and hence transported even over relatively large distances; in soil, however, efficient transport might be limited to particles of smaller size.

Additionally, collembolans seem to be actively involved in the production of microparticles from bigger chunks of biochar by grazing activities (e.g. [19]) that result in ingestion of microparticles, as we observed in terms of biochar particles in the gut of many individuals. This feeding presumably would result in defecation in another place. Presumably, ingestion/defecation might only be important for particles smaller than 100μm, but the abundance of mesofauna in soils suggests that transport might be substantial and should be considered in future studies.

*Folsomia candida* transported the three biochar types at different frequencies (with preference for biochar based on pine bark). It is primarily a fungal hypha grazer with strong preferences for particular species ([27]). Most likely our observations are the result of the different abiotic characteristics ([16]) of the respective biochar types resulting in differing microbial communities on the surface (e.g. [18]; [15]; [5]). The microbial diversity might be increased in presence of biochar ([17]; [15]), however, bacteria and fungi react differently to changes e.g. in pH ([33]; [34]; [35]). Other reasons may relate to particle shape or surface characteristics (i.e., how readily particles accumulate on the springtail body), however, biochar can also be used as a food source by Collembola as Ding et al. ([36]) report that organic components can be of nutritional advantage for the respective symbiotic gut bacteria. Additionally, the passage through the gut could presumably enhance the decomposition of the biochar by an inoculation with bacteria which might be able to survive outside the gut on the feces ([37]) and hence potentially modify the soil’s microbial community to some extent ([38])

Our study shows that microarthropods can be involved in the horizontal transport of biochar particles. This transport could be quite important for spreading biochar particles from the locations to which they were applied. Horizontal, and perhaps vertical ([39]), transport can thus contribute to explaining the exposure of soil biota to biochar particles on a local scale. Additionally, the distribution of biochar particles to deeper soil layers presumably has consequences for the interaction of these particles with soil minerals and hence soil fertility ([40]). Future studies should involve testing the transport of biochar particles of different sizes in soil to get a more detailed understanding of the interaction with soil organisms and potential ecotoxicology ([41]; [26]) over time ([15]).

## Supporting information

S1 File Original data table. (DOCX)
