# Supplementary material for: Collembola laterally move biochar particles
Source: PLoS One. 2019 Nov 1;14(11):e0224179. doi: 10.1371/journal.pone.0224179 (PMC6824558; doi:10.1371/journal.pone.0224179)
Supplement: S1 File — (DOCX) [file pone.0224179.s001.docx]

**Supporting Information**

S1: Original data table, given are: sample ID, Treatment (*Folsomia candida* vs control, the latter containing no *F. candida*), Biochar type (SH = Spelt husks; PB = Pine bark, PW = Pine wood), T0 = day of setup of experiment; T10 = day 10 after setup of experiment. Numbers are n of particles in the respective ring (with ring 1 = 1 cm, ring 2 = 2 cm, ring 3 = 3 cm and ring 4 = 4 cm diameter around central feeding station).

| sample ID | Treatment | Biochar type | T0 | | | | T10 | | | |
| --- | --- | --- | --- | --- | --- | --- | --- | --- | --- | --- |
|  |  |  | Ring1 | Ring2 | Ring3 | Ring4 | Ring1 | Ring2 | Ring3 | Ring4 |
| 1 | *Folsomia candida* | SH | 3 | 5 | 5 | 4 | 45 | 14 | 16 | 9 |
| 2 | *Folsomia candida* | SH | 12 | 14 | 13 | 15 | 113 | 98 | 89 | 56 |
| 3 | *Folsomia candida* | SH | 13 | 6 | 12 | 14 | 42 | 38 | 43 | 37 |
| 4 | *Folsomia candida* | SH | 7 | 3 | 5 | 8 | 71 | 15 | 36 | 29 |
| 5 | *Folsomia candida* | SH | 8 | 9 | 12 | 13 | 30 | 36 | 32 | 29 |
| 6 | *Folsomia candida* | SH | 5 | 6 | 10 | 9 | 19 | 12 | 10 | 11 |
| 7 | *Folsomia candida* | SH | 3 | 9 | 9 | 13 | 28 | 41 | 39 | 54 |
| 8 | *Folsomia candida* | SH | 6 | 6 | 8 | 9 | 124 | 63 | 78 | 81 |
| 9 | *Folsomia candida* | PB | 7 | 9 | 10 | 15 | / | / | / | / |
| 10 | *Folsomia candida* | PB | 8 | 10 | 13 | 7 | 472 | 187 | 124 | 134 |
| 11 | *Folsomia candida* | PB | 9 | 11 | 14 | 12 | 223 | 112 | 91 | 114 |
| 12 | *Folsomia candida* | PB | 12 | 15 | 16 | 15 | 489 | 195 | 143 | 130 |
| 13 | *Folsomia candida* | PB | 6 | 5 | 3 | 5 | 215 | 161 | 82 | 99 |
| 14 | *Folsomia candida* | PB | 9 | 13 | 7 | 9 | 239 | 149 | 87 | 123 |
| 15 | *Folsomia candida* | PB | 11 | 6 | 12 | 10 | 248 | 125 | 102 | 130 |
| 16 | *Folsomia candida* | PB | 4 | 7 | 8 | 11 | 285 | 82 | 85 | 118 |
| 17 | *Folsomia candida* | PW | 8 | 15 | 17 | 14 | 132 | 77 | 65 | 66 |
| 18 | *Folsomia candida* | PW | 18 | 11 | 15 | 16 | 145 | 81 | 69 | 52 |
| 19 | *Folsomia candida* | PW | 11 | 7 | 9 | 8 | 125 | 49 | 54 | 69 |
| 20 | *Folsomia candida* | PW | 12 | 10 | 11 | 17 | 250 | 97 | 79 | 92 |
| 21 | *Folsomia candida* | PW | 10 | 6 | 7 | 10 | 213 | 104 | 80 | 94 |
| 22 | *Folsomia candida* | PW | 6 | 8 | 9 | 11 | 220 | 109 | 94 | 96 |
| 23 | *Folsomia candida* | PW | 10 | 12 | 10 | 15 | 173 | 95 | 86 | 79 |
| 24 | *Folsomia candida* | PW | 11 | 19 | 17 | 18 | 187 | 98 | 104 | 91 |
| 25 | Control | SH | 0 | 0 | 0 | 0 | 0 | 0 | 0 | 0 |
| 26 | Control | SH | 0 | 0 | 0 | 0 | 0 | 0 | 0 | 0 |
| 27 | Control | SH | 0 | 0 | 0 | 0 | 0 | 0 | 0 | 0 |
| 28 | Control | SH | 0 | 0 | 0 | 0 | 0 | 0 | 0 | 0 |
| 29 | Control | SH | 0 | 0 | 0 | 0 | 0 | 0 | 0 | 0 |
| 30 | Control | SH | 0 | 0 | 0 | 0 | 0 | 0 | 0 | 0 |
| 31 | Control | SH | 0 | 0 | 0 | 0 | 0 | 0 | 0 | 0 |
| 32 | Control | SH | 0 | 0 | 0 | 0 | 0 | 0 | 0 | 0 |
| 33 | Control | PB | 0 | 0 | 0 | 0 | 0 | 0 | 0 | 0 |
| 34 | Control | PB | 0 | 0 | 0 | 0 | 0 | 0 | 0 | 0 |
| 35 | Control | PB | 0 | 0 | 0 | 0 | 0 | 0 | 0 | 0 |
| 36 | Control | PB | 0 | 0 | 0 | 0 | 0 | 0 | 0 | 0 |
| 37 | Control | PB | 0 | 0 | 0 | 0 | 0 | 0 | 0 | 0 |
| 38 | Control | PB | 0 | 0 | 0 | 0 | 0 | 0 | 0 | 0 |
| 39 | Control | PB | 0 | 0 | 0 | 0 | 0 | 0 | 0 | 0 |
| 40 | Control | PB | 0 | 0 | 0 | 0 | 0 | 0 | 0 | 0 |
| 41 | Control | PW | 0 | 0 | 0 | 0 | 0 | 0 | 0 | 0 |
| 42 | Control | PW | 0 | 0 | 0 | 0 | 0 | 0 | 0 | 0 |
| 43 | Control | PW | 0 | 0 | 0 | 0 | 0 | 0 | 0 | 0 |
| 44 | Control | PW | 0 | 0 | 0 | 0 | 0 | 0 | 0 | 0 |
| 45 | Control | PW | 0 | 0 | 0 | 0 | 0 | 0 | 0 | 0 |
| 46 | Control | PW | 0 | 0 | 0 | 0 | 0 | 0 | 0 | 0 |
| 47 | Control | PW | 0 | 0 | 0 | 0 | 0 | 0 | 0 | 0 |
| 48 | Control | PW | 0 | 0 | 0 | 0 | 0 | 0 | 0 | 0 |
